# Ankle-foot orthoses in children with cerebral palsy: a cross sectional population based study of 2200 children

**DOI:** 10.1186/1471-2474-15-327

**Published:** 2014-10-02

**Authors:** Maria Wingstrand, Gunnar Hägglund, Elisabet Rodby-Bousquet

**Affiliations:** Department of Clinical Sciences, Lund, Orthopedics, Lund University, Lund, S-221 85 Sweden; Department of Orthopedics, Helsingborg Hospital, Helsingborg, S-251 87 Sweden; Centre for Clinical Research, Uppsala University, Central Hospital, Västerås, S-721 89 Sweden

**Keywords:** Ankle-foot orthosis, AFO, Cerebral palsy, Range of motion, Total population

## Abstract

**Background:**

Ankle-foot orthosis (AFO) is the most frequently used type of orthosis in children with cerebral palsy (CP). AFOs are designed either to improve function or to prevent or treat muscle contractures. The purpose of the present study was to analyse the use of, the indications for, and the outcome of using AFO, relative to age and gross motor function in a total population of children with cerebral palsy.

**Methods:**

A cross-sectional study was performed of 2200 children (58% boys, 42% girls), 0–19 years old (median age 7 years), based on data from the national Swedish follow-up programme and registry for CP. To analyse the outcome of passive ankle dorsiflexion, data was compared between 2011 and 2012. The Gross motor classification system (GMFCS) levels of included children was as follows: I (*n* = 879), II (*n* = 357), III (*n* = 230), IV (*n* = 374) and V (*n* = 355).

**Results:**

AFOs were used by 1127 (51%) of the children. In 215 children (10%), the indication was to improve function, in 251 (11%) to maintain or increase range of motion, and 661 of the children (30%) used AFOs for both purposes. The use of AFOs was highest in 5-year-olds (67%) and was more frequent at lower levels of motor function with 70% at GMFCS IV-V. Physiotherapists reported achievement of functional goals in 73% of the children using AFOs and maintenance or improvement in range of ankle dorsiflexion in 70%.

**Conclusions:**

AFOs were used by half of the children with CP in Sweden. The treatment goals were attained in almost three quarters of the children, equally at all GMFCS levels. AFOs to improve range of motion were more effective in children with a more significant decrease in dorsiflexion at baseline.

**Electronic supplementary material:**

The online version of this article (doi:10.1186/1471-2474-15-327) contains supplementary material, which is available to authorized users.

## Background

Cerebral palsy (CP) with a prevalence of about 2.2/1000 live births is the most common cause of motor disability in childhood [1]. Spasticity, muscle weakness, impaired postural control, and selective motor control are some of the primary manifestations of this brain injury [2]. As a consequence, children with CP are at increased risk of developing secondary complications such as muscle contractures, skeletal deformities, hip dislocation, and scoliosis [3]. Two thirds of children with CP walk with or without walking aids [4, 5]. However, children with CP usually start to walk later than their peers without CP [6], walk at a slower speed, and with a higher energy cost [7].

Orthoses are widely used in the management of children with CP even though there is no robust evidence for its effect [8, 9]. The ankle-foot orthosis (AFO) is the most frequently used type of orthoses in the lower limb [10]. AFOs are designed to address two main concerns; either to improve function or to affect the body structure (i.e. to prevent or treat contractures). AFOs have been found to increase walking speed and reduce the energy cost in children with CP [11]. In Sweden, orthoses are provided free of charge to the families of the children who need them. Orthoses are prescribed by a physician, often on the recommendation from the child´s physiotherapist or occupational therapist.

CPUP is a secondary prevention follow-up programme and health care quality registry that was introduced in southern Sweden in 1994. Since 2005, all regions in Sweden offer participation in the programme [12, 13]. In southern Sweden, 98% of people with CP born 1990 and thereafter participate and from birth year 2000 about 95% of all people with CP in all parts of Sweden participate. In CPUP, children are examined regularly by their physiotherapist and the use of orthoses and the indications for treatment are recorded. However, treatment is decided upon by the child’s physiotherapist and physician according to clinical signs and local routines.

Our purpose was to analyse the use of, the indications for, and the treatment goals of using AFO relative to age and gross motor function in a total population of children with CP.

## Methods

We performed a cross-sectional study based on data from CPUP on all children with CP born 1991 to 2011 who were reported into the registry in 2011 (Table 1). The adolescents born 1991–1997 represent the population in two regions in southern Sweden with a population of 1.4 million. Those born 1998–1999 represent an additional seven regions with a population of 5.0 million. The children born 2000–2011 represent all 21 regions in Sweden with a total population of 9.4 million. Children below the age of four with presumed, although not yet confirmed, CP are included in CPUP, and also in the present study (*n* = 518). From previous experience approximately 2% of the children enrolled in CPUP at an early age have their diagnosis rejected at the age of 4–5 years and are excluded from the registry. Children in the CPUP registry who had undergone gastrosoleus, tendo achilles lengthening or foot surgery for varus or valgus deformity (*n* = 227) were also included.

**Table 1:** **Details in 2011 of the 2200 participants aged 0**–**19 years**

	All participants						Southern region		All other regions	
Year of birth	1991-2011		1991-1999		2000-2011		2000-2011		2000-2011	
	n	%	n	%	n	%	n	%	n	%
**Gender**										
Girls	929	42%	147	41%	782	43%	127	42%	655	43%
Boys	1271	58%	211	59%	1060	58%	177	58%	883	57%
**GMFCS**										
I	879	40%	120	34%	759	41%	93	31%	666	43%
II	357	16%	71	20%	286	16%	61	20%	225	15%
III	230	11%	38	11%	192	10%	44	15%	148	10%
IV	374	17%	66	18%	308	16%	54	18%	254	17%
V	355	16%	63	18%	292	17%	51	17%	241	16%
Unclassified	5		0		5		1		4	
**AFO**										
AFO users	1127	51%	148	41%	979	53%	156	51%	823	54%
For function	215	10%	29	8%	186	10%	27	9%	159	10%
For function & ROM	661	30%	87	24%	574	31%	94	31%	480	31%
For ROM	251	11%	32	9%	219	12%	35	11%	184	12%

Comparison of different cohorts relative to year of birth and regions included.

The CP diagnosis was determined by a neuropediatrician at the age of four. Exclusion and inclusion criteria were in accordance with the Surveillance of Cerebral Palsy network in Europe, SCPE [14]. The CPUP programme includes a continuous standardized follow-up of gross motor function, treatment, and clinical findings, including radiographic examinations of hips and spine. Gross motor function is classified by a physiotherapist according to the expanded and revised version of the Gross Motor Function Classification System (GMFCS), which is an age-related system with five levels ranging from I to V where the child with level V is the most affected [15]. Passive range of joint motion is measured with a goniometer in a standardized position according to a manual (http://www.cpup.se).

In CPUP, AFOs include all types of orthoses that proximally ends below the knee joint and extends distally over the foot. The use of AFOs to improve function or to maintain/increase range of dorsiflexion is reported separately. AFOs for function are divided into: 1) Improve walking ability; 2) Improve balance/stability; or 3) Facilitate training. AFOs can also be reported for “other purposes”. Whether or not the goal of using the AFOs was attained in these specific areas is reported by the physiotherapist as “yes” or ”no”.

When AFOs are used to maintain/increase range of motion, treatment time is reported by families and therapists. To analyse the outcome of the AFO-treatment for range of motion the differences in passive ankle dorsiflexion with the knee extended, including gastrocnemius, were calculated between the examinations in 2011 and 2012 for those reported to use AFO both years. In all analyses, the leg with the poorest development was recorded in those treated bilaterally, and the leg with AFO in the children treated unilaterally. The material was analysed separately for the two southern regions in Sweden and the rest of Sweden for those born 2000–2011 to reveal any differences between the cohorts. Data concerning AFO were passively recorded in CPUP and no recommendations were given concerning AFO to local clinicians based upon the data entered. The choices for orthotic use are made by the local physiotherapist and clinician.

The study was approved by the Medical Research Ethics Committee at Lund University (LU-443-99), and permission was obtained to extract data from the CPUP registry.

### Statistical analyses

Chi-square and Z-test comparison of column proportions after Bonferroni adjusted *p*-values were used to analyse differences between children born 2000–2011 representing different geographical areas of Sweden. Nonparametric tests such as Chi-square, Chi-square for trends (Linear-by linear association test), and Spearman’s rank correlation test (*r*^*s*^) were used to analyse the relationships between variables. For the statistical analysis of range of motion related to the time AFOs are worn per day, the answers were grouped into three intervals, 4 hours or less, 5 to 6 hours, and 7 hours or more. The Wilcoxon Rank Sum test was used to analyse the medians of baseline range of motion between those with decreased, maintained and improved range of motion 2012 compared to 2011. SPSS version 21.0 and R software environment were used for the statistical analyses and *p*-values less than 0.05 were considered significant.

## Results

In total, 2200 children and adolescents 0 to 19 years old (median age 7 years) were recorded in the registry in 2011. Of those 1271 (58%) were boys and 929 (42%) were girls. The distribution of children in relation to gender, age and levels of GMFCS is presented in Table 1. The distribution was similar in the cohorts from different regions in Sweden for those born 2000–2011 (Table 1).

In 2011, AFOs were used by 1127 (51%) of the children with similar percentages for boys (52%) and girls (51%). In 215 children (10%) the indication was to improve function, in 251 (11%) to maintain or increase range of motion, and 661 of the children (30%) used AFOs for both purposes (Table 1). For children born 2000–2011 there were no significant differences between the different cohorts in southern Sweden compared to the rest of Sweden regarding the use of AFOs (*p* = 0.483) and the indication for wearing AFOs (*p* = 0.572-0.812) (Table 1).

**Figure 1:**
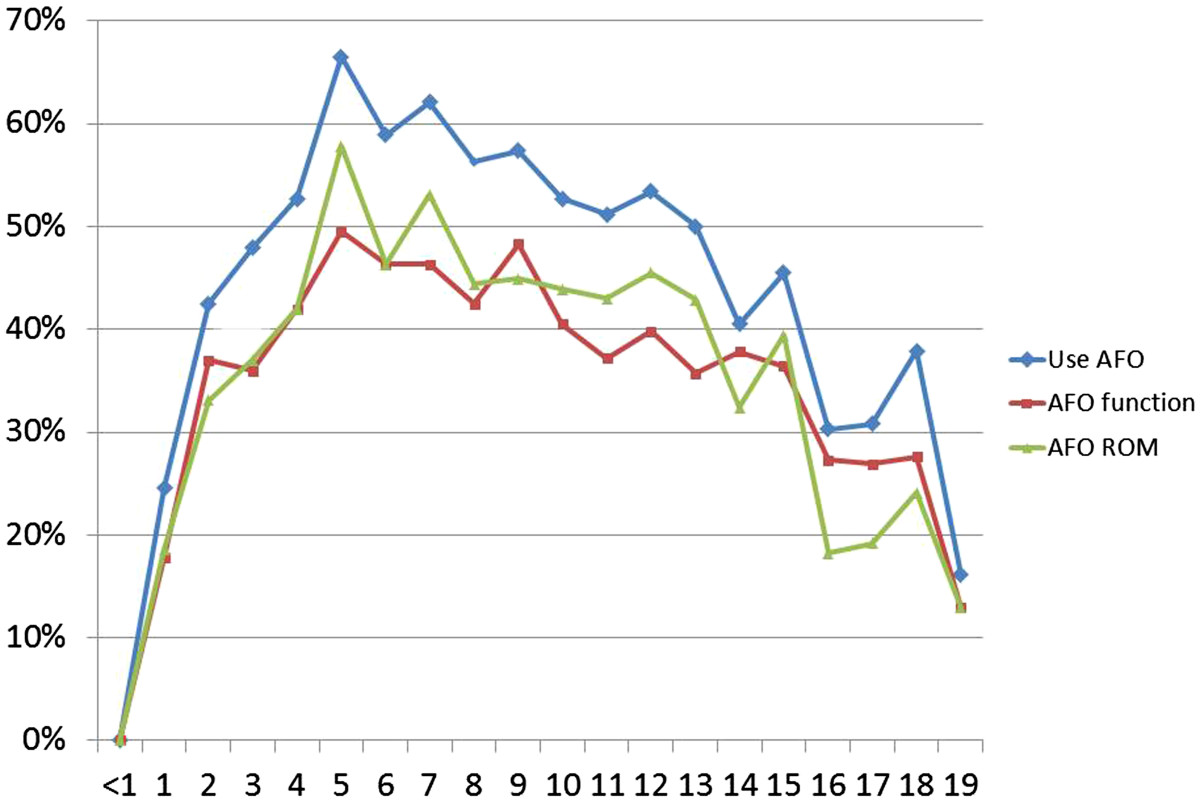
**Use of ankle**-**foot orthosis** **(** **AFO** **)** **related to age in 2200 children with CP.** The proportion of children (%) in each age group indicated for the total material and for children treated to improve function and joint range of motion (ROM) respectively.

The 2011 dataset showed an increasing trend (*p* < 0.001) in the use of AFO relative to the levels of gross motor function, from 34% in children at GMFCS I to approximately 70% at GMFCS IV and V (Table 2). AFOs to improve function were mostly used by children at GMFCS level IV (63%), while AFOs to maintain/increase ankle dorsiflexion were most frequent in children at GMFCS level V (61%). The use of AFOs increased with age to 67% in 5-year olds and then declined to 16% in 19-year olds (Figure 1). The same development was seen for both genders irrespective of indication for AFO.

**Table 2:** **Number of children using Ankle**-**foot orthosis** (**AFO**) **to improve function or range of motion** (**ROM**) **relative to GMFCS levels I**-**V**

	GMFCS I		GMFCS II		GMFCS III		GMFCS IV		GMFCS V		Missing	Total	
	n = 879		n = 357		n = 230		n = 374		n = 355				
	nt (%)	ng (%)	nt (%)	ng (%)	nt (%)	ng (%)	nt (%)	ng (%)	nt (%)	ng (%)		nt (%)	ng (%)
AFO total	299 (34)		168 (47)		148 (64)		267 (71)		243 (69)		2	1127 (51)	
AFO function	183 (21)		138 (39)		127 (55)		237 (63)		189 (53)		2	876 (40)	
Walking ability	159 (18)	102 (64)	123 (35)	84 (68)	106 (46)	79 (75)	122 (33)	91 (75)	0 (0)	0 (0)	2	512 (23)	358 (70)
Balance, stability	110 (13)	76 (69)	115 (32)	82 (71)	115 (50)	90 (78)	204 (55)	159 (78)	129 (36)	99 (77)	2	675 (31)	503 (75)
Facilitate training	15 (2)	11 (73)	23 (7)	16 (70)	42 (18)	35 (83)	81 (22)	59 (73)	71 (20)	51 (72)	1	233 (11)	172 (74)
AFO other purpose	32 (4)	15 (47)	17 (5)	12 (70)	12 (5)	11 (92)	32 (9)	25 (78)	53 (15)	43 (81)	0	146 (7)	105 (72)
AFO ROM 2011	254 (29)		129 (36)		105 (46)		207 (55)		216 (61)		1	912 (41)	
AFO ROM 2011-2012	148 (17)	107 (72)	87 (24)	56 (64)	83 (36)	56 (68)	144 (39)	107 (74)	155 (44)	110 (71)		617 (28)	436 (71)

Nt = total number of children using AFO for each purpose in percent (%) of all 2200 children with CP; ng = number of children who attained the goal in percent (%) of those using AFO for each purpose.

Several children attained more than one goal.

Of the 876 children wearing AFOs for function in 2011, many attained more than one goal. In 512 children who used AFO to improve walking, 70% attained the goal (Table 2). AFOs to improve balance/stability were reported for 675 children, 75% of the children reached this goal. AFOs to facilitate training were used by 233 children, and of those 74% attained the goal. Of the 146 children using AFOs for “other” reasons such as postoperative immobilization etc., 73% reached the goal. All children reported to use AFO for other purposes also used them for function and/or range of motion. In summary, 73% of the children wearing AFOs to improve function reached one or several goals. The goal attainment correlated significantly (*p* < 0.001) to the purpose of the AFO, such as improved walking ability (*r*^*s*^ = 0.77), improved balance/stability (*r*^*s*^ = 0.80), facilitated training (*r*^*s*^ = 0.84), or other purposes (*r*^*s*^ = 0.84).

**Figure 2:**
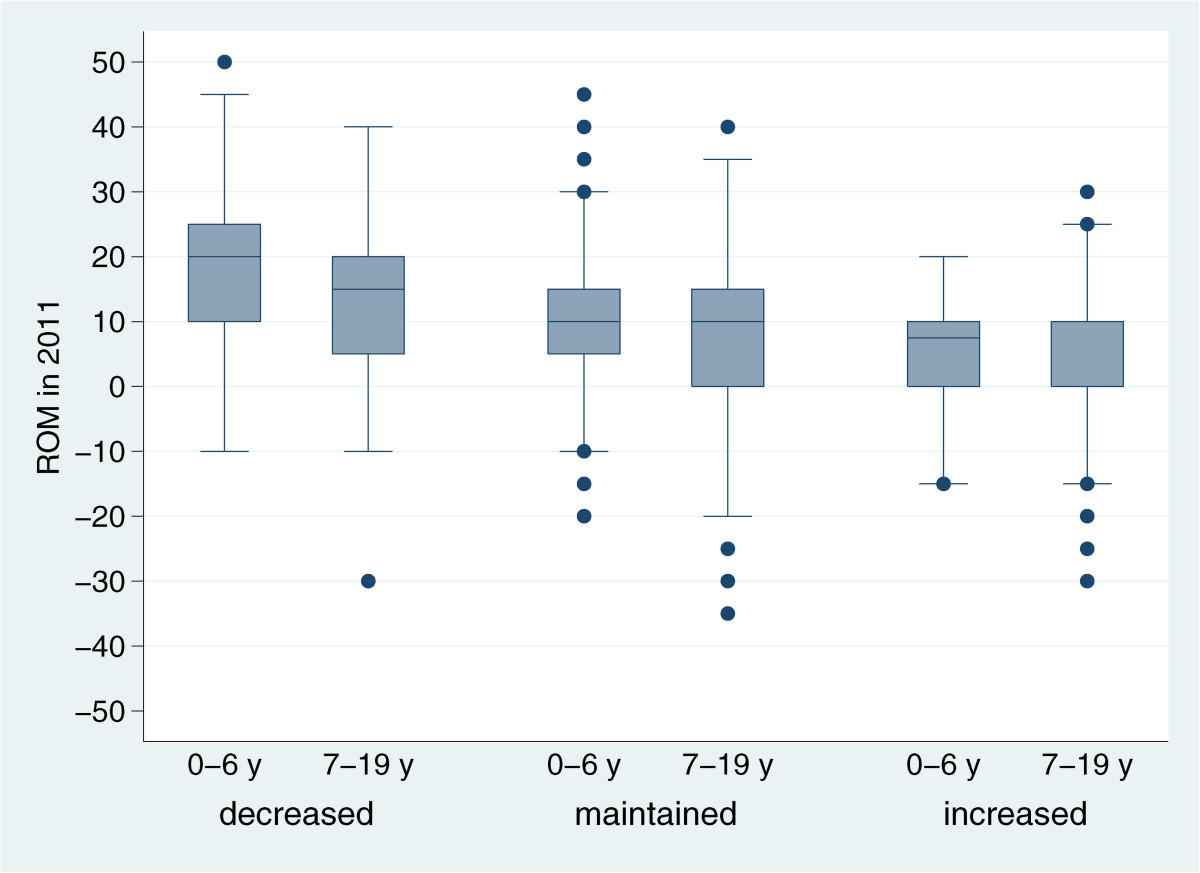
**Boxplot presenting the range of ankle dorsiflexion at baseline in 2011,** **for 617 children using ankle**-**foot orthoses** **(** **AFOs** **)** **in 2011 and 2012,** **to maintain or improve range of motion (ROM).** *Decreased* = children with >5 degrees decrease in dorsiflexion 2012 compared to 2011, *Maintained* = children with no change +/-5 degrees in dorsiflexion 2012, *Increased* = children with >5 degrees increase in dorsiflexion 2012. Children are divided into age-groups 0–6 years (*n* = 338) and 7–19 years old (*n* = 279).

Of the 912 children using AFOs to maintain or improve ankle dorsiflexion in 2011, 259 (28%) used orthoses 4 hours or less, 172 (19%) 5–6 hours, and 481 (53%) children used the AFOs 7 hours or more per day. There were 617 children who used AFOs to maintain or improve ROM both in 2011 and in 2012, and for whom information of range of passive ankle dorsiflexion and treatment time were reported both years. Of these 617 children, 181 (29%) showed decreased ROM with >5 degrees in 2012, 380 (62%) maintained the same ROM +/- 5 degrees, and 56 (9%) improved their ROM with >5 degrees. Nine of the children were treated with gastrosoleus and/or foot surgery between the measurements in 2011 and 2012. Out of these children 3 showed decreased, 3 maintained and 3 improved ROM. The proportion who maintained or improved their ankle dorsiflexion was similar (*p* = 0.579) in children at all GMFCS levels (Table 2); i.e. 60% at GMFCS level I and 61.5% at GMFCS V maintained their ROM, while 12% at GMFCS I and 10% at level V improved their dorsiflexion. There was no statistically significant difference in goal attainment related to the time the AFOs were used/day. The difference in outcome was based on the initial range of motion, where children who maintained or improved their ROM had a more significant decrease in ROM at baseline (*p* < 0.001) (Figure 2). Of the younger children aged 0–6 years 66% maintained or improved their ROM compared to 76% of the 7–19 year old children using AFO, implying a significant difference (*p* = 0.005) related to age. This may be explained by a slightly higher ROM at baseline in the younger children (median 10°; range -20°/50°; percentiles 25/75 = 5°/20°) than in the older children (median 10°; range -35°/40°; percentiles 25/75 = 0°/15°) as in Figure 2. There was no significant difference in daily treatment time between the three groups. Of the 518 children who were below the age of 4 years in 2011, 207 used AFO and of these only two children (1%) had their CP-diagnosis rejected.

## Discussion

There is little evidence in the literature of the effectiveness of AFOs in children with CP, and the variability in different studies makes it difficult to compare the results [8, 16, 17]. This is a cross-sectional study of the use of AFO in children with CP, based on data from the CPUP registry. As such, it provides unique data for a total population of children at all GMFCS levels. However, this study has several limitations and one is that data partly represent different cohorts, the older children representing the southern regions of Sweden and the younger children representing the whole Sweden. There is also a lack of information regarding materials, foot position, gait patterns and in what activities the AFO is worn to evaluate different types of AFO in more detail. Another limitation is that treatment time was reported by the caregivers, and that goal attainment for function is entirely based on information from the child’s physiotherapist and the families. Finally, this is a registry study representing a total population of children with CP in Sweden. All children are treated by their local team and additional treatments to reduce muscle tone, improve function and ROM are not controlled for. The nine children who had undergone gastrosoleus lengthening or foot surgery for varus or valgus deformity during follow-up corresponds to 1.4% of all 617 children. Their results were distributed equally and not likely to have affected the results.

Orthotics for children such as AFOs are provided free of charge in Sweden so the results are independent of the socioeconomic situation of the families. In the CPUP-programme all children are followed with repeated standardized examinations including measurements of passive joint range of motion. As a result, contractures are often detected at an early stage and can be treated non-operatively [12]. Consequently, the proportion of children treated with AFOs (51%) could be higher than in areas without a prevention programme.

The indication for AFO was to improve function in 40% of all children with CP. Brehm et al. have shown that AFOs reduce the energy cost of walking in children with CP [11]. In the present study 70% of those using AFOs to improve walking ability attained the goal. Most of the children using AFOs but not fulfilling the functional goal used their orthoses also with the indication to maintain or improve range of ankle dorsiflexion. In non-walking children, AFOs for function were mainly used to improve balance and stability which is essential in standing and sitting positions.

The use of AFOs to maintain or improve ankle dorsiflexion was reported in 41% of all children. A study by Tardieu et al. [18] concluded that the soleus muscle must be stretched at least six hours per day to prevent contracture development. In the present study more than half of the children who wore AFOs to improve range of motion used orthoses 7 hours or more. However, this study could not reveal any statistically significant differences in goal attainment related to the treatment time. Children who maintained or improved their dorsiflexion had a lower range of motion at baseline compared with those who decreased their dorsiflexion (Figure 2). This is of clinical importance since the children with more limited range of motion appears to benefit the most from the AFO treatment. AFOs to maintain or improve ROM were used by children at all GMFCS levels, and goal attainment was just as high in children without walking ability as in walking children. Children at higher levels of motor function sometimes use AFOs to improve ROM and thereby improve gait. In children at lower levels of motor function AFOs are frequently used from an early age to keep the feet in a neutral position and prevent the development of deformities. This is of clinical importance to increase the base of support, and provide stability in both sitting and standing positions, and reduce the risk of pressure sores and pain. AFOs can also be used to stabilize the joint and prevent an excessive range of motion. We know from a previous study based on children in CPUP from southern Sweden [19] that ankle dorsiflexion, measured with flexed knee, increase in children in GMFCS V from 23 (95% CI 23–26) degrees at four years of age to 28 (95% CI 23–33) degrees at 14 years of age. By reducing the number of achilles lengthenings and sometimes by AFO treatment some of these calcaneus feet are probably prevented.

The outcome also seem to be age-related where a higher proportion of 7–19 year old children maintained or improved their range of motion when treated with AFO compared to 0–6 year old children. This may be explained by their initial lower ROM. The use of AFOs peaked at 5 years of age. In two earlier studies based on children from southern Sweden, the development of spasticity of the gastrosoleus muscle and range of motion of the ankle joint with age were analysed [19, 20]. The muscle tone, measured with the Modified Ashworth scale, [21] increased until 4–6 years of age and then decreased at 12–15 years of age [20]. The mean range of dorsiflexion of the foot decreased from 30° to 20° up to 5 years of age and then persisted at the same level for the remaining growth period [19]. These findings may explain why 4–6 year old children presented the highest use of AFOs.

## Conclusions

Half of all children with CP in Sweden use AFOs to improve function and/or to maintain or improve range of motion. The use of AFO is most frequent at 4–6 years of age and in children with lower levels of gross motor function. In this study, three quarters of the children treated with AFO attained the treatment goals, i.e. improved function and/or maintained/improved range of motion. A higher proportion of the children with a lower range of motion at baseline improved their ankle dorsiflexion using AFOs compared to children with a higher initial range of motion.

## Authors’ original submitted files for images

Below are the links to the authors’ original submitted files for images. Authors’ original file for figure 1 Authors’ original file for figure 2

## Abbreviations

AFO: Ankle-foot orthosis
CP: Cerebral palsy
CPUP: Cerebral palsy follow up programme and quality registry
GMFCS: Gross motor function classification system
ROM: Joint range of motion.
