# Supplementary figures and images for: Ankle-foot orthoses in children with cerebral palsy: a cross sectional population based study of 2200 children
Source: BMC Musculoskelet Disord. 2014 Oct 2;15:327. doi: 10.1186/1471-2474-15-327 (PMC4192348; doi:10.1186/1471-2474-15-327)

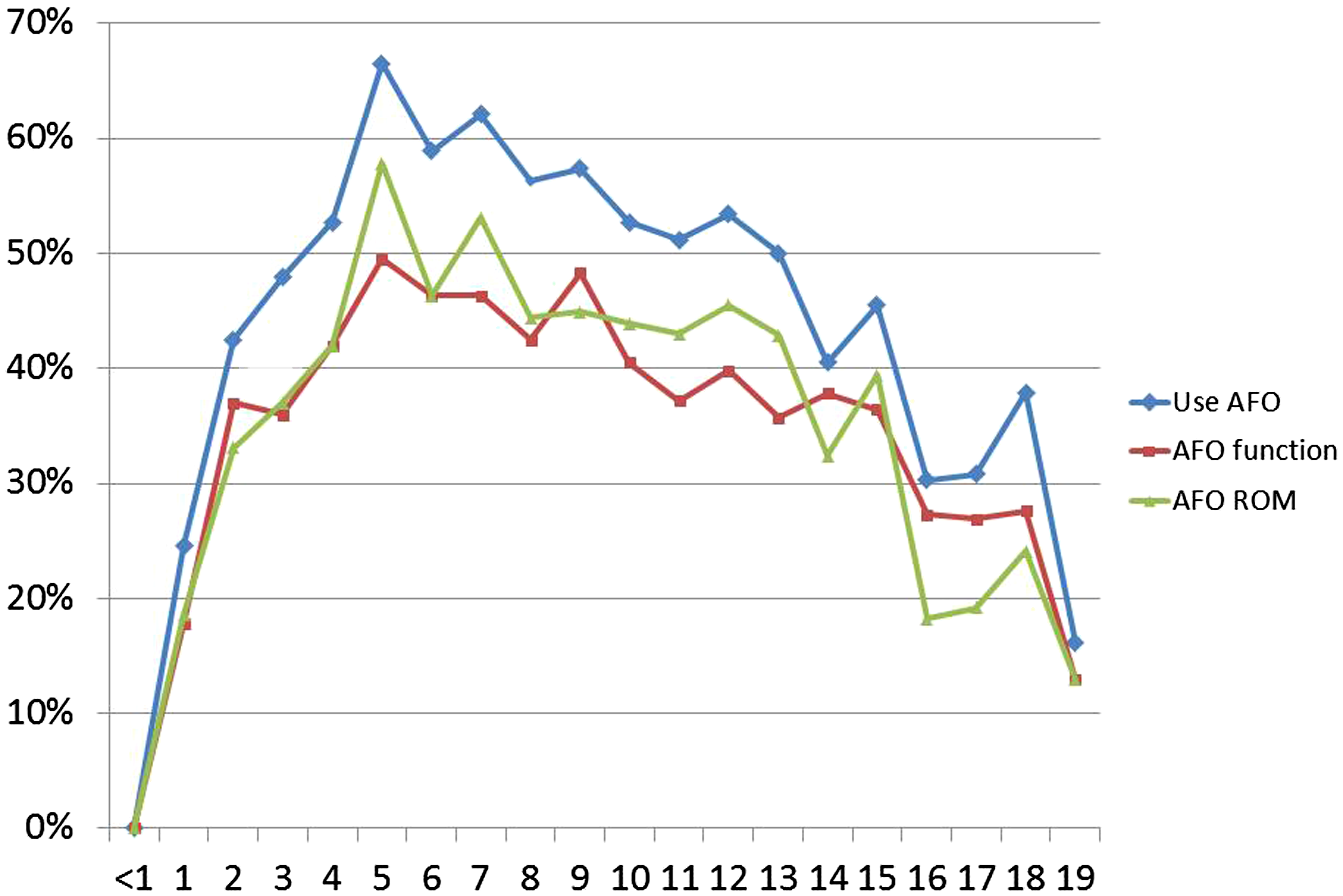

Supplement: Supplementary file 1 — Authors’ original file for figure 1 [file 12891_2014_2262_MOESM1_ESM.tif]

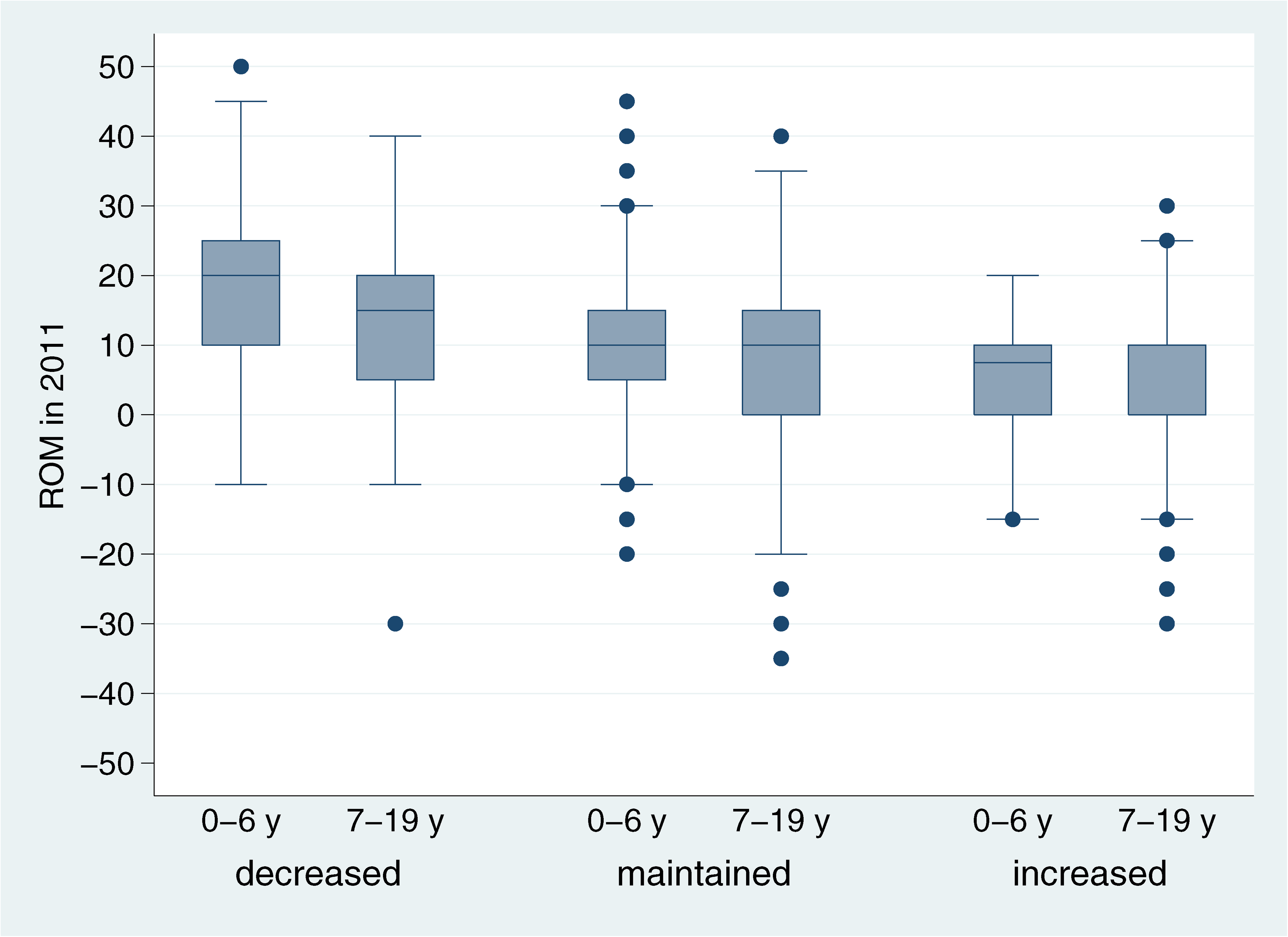

Supplement: Supplementary file 2 — Authors’ original file for figure 2 [file 12891_2014_2262_MOESM2_ESM.tif]
